# Perceptions of Readiness for Practice After Complex General Surgical Oncology Fellowship: A Survey Study

**DOI:** 10.1245/s10434-023-14524-x

**Published:** 2023-11-07

**Authors:** Shay Behrens, Heather A. Lillemoe, Sean P. Dineen, Maria C. Russell, Brendan Visser, Russell S. Berman, Jeffrey M. Farma, Elizabeth Grubbs, Jeremy L. Davis

**Affiliations:** 1 Surgical Oncology Program, Center for Cancer Research, National Cancer Institute, Bethesda, MD USA; 2 Department of Surgical Oncology, The University of Texas MD Anderson Cancer Center, Houston, TX USA; 3 Department of Gastrointestinal Oncology, H. Lee Moffitt Cancer Center, Tampa, FL USA; 4 Division of Surgical Oncology, Winship Cancer Institute, Emory University, Atlanta, GA USA; 5 Department of General Surgery, Stanford Hospital, Stanford, CA USA; 6 Division of Surgical Oncology, Department of Surgery, New York University Grossman School of Medicine, New York, NY USA; 7 Department of Surgical Oncology, Fox Chase Cancer Center, Philadelphia, PA USA

## Abstract

**Background:**

Surgical subspecialty training aims to meet the needs of practicing surgeons and their communities. This study investigates career preparedness of Complex General Surgical Oncology (CGSO) fellowship graduates, identifies factors associated with practice readiness, and explores potential opportunities to improve the current training model.

**Methods:**

The Society of Surgical Oncology partnered with the National Cancer Institute to conduct a 36-question survey of CGSO fellowship graduates from 2012 to 2022.

**Results:**

The overall survey response rate was 38% (221/582) with a slight male predominance (63%). Forty-six percent of respondents completed their fellowship after 2019. Factors influencing fellowship program selection include breadth of cancer case exposure (82%), mentor influence (66%), and research opportunities (38%). Overall, graduates reported preparedness for practice; however, some reported unpreparedness in research (18%) and in specific clinical areas: thoracic (43%), hyperthermic intraperitoneal chemotherapy (HIPEC) (15%), and hepato-pancreato-biliary (15%) surgery. Regarding technical preparedness, 70% reported being “very prepared”. Respondents indicated lack of preparedness in robotic (63%) and laparoscopic (33%) surgery approaches. Suggestions for training improvement included increased autonomy and case volumes, program development, and research infrastructure. Current practice patterns by graduates demonstrated discrepancies between ideal contracts and actual practice breakdowns, particularly related to the practice of general surgery.

**Conclusions:**

This study of CGSO fellowship graduates demonstrates potential gaps between trainee expectations and the realities of surgical oncology practice. Although CGSO fellowship appears to prepare surgeons for careers in surgical oncology, there may be opportunities to refine the training model to better align with the needs of practicing surgical oncologists.

**Supplementary Information:**

The online version contains supplementary material available at 10.1245/s10434-023-14524-x.

The development of a complex general surgical oncology (CGSO) fellowship was driven by the increasing complexity of cancer cases and the need for specialized training to provide high-quality and multidisciplinary care to cancer patients. Although its origins can be traced back to the 1930s, surgical oncology was formally established as a specialty in 1975.^1^ CGSO fellowships were officially approved and sponsored by the Society of Surgical Oncology in 1983, and the formal CGSO American Board of Surgery certification was recently introduced in 2011.^2,3^

In recent years, there has been a slight downtrend in the number of applicants to CGSO fellowships.^4^ An increase in subspeciality fellowships that overlap clinically with the CGSO fellowship, such as breast surgical oncology, surgical endocrinology, colon and rectal surgery, and hepato-pancreato-biliary (HPB) surgery may be contributing to this phenomenon.^5,6^ Although subspecialized training has led to improved patient satisfaction and survival rates amongst those treated by specialists, such as breast surgeons, specialized training may limit a surgeon’s scope of practice and breadth of expertise.^7^ Furthermore, a recent 2019 survey study highlighted the clinical practice patterns of CGSO graduates, finding that most graduates return to their hometown or previous training institutions, or both.^8^ These patterns may result in concentrated areas of practicing surgical oncologists in larger, urban cities, leaving a greater need in rural and underserved areas. Despite the concentration in large, urban cities, the authors found that most graduates maintain a broad-based practice, and only 27% of graduates focus exclusively on one disease site. The need for specialized surgeons, the shortage of rural surgeons, and the overlap with other fellowships create unique challenges in determining the direction of the CGSO fellowship.

In addition, the effectiveness of CGSO fellowship in preparing one for a career in surgical oncology is not fully understood due to the lack of publicly available data specific to this issue. We sought to gauge the perceptions of recent CGSO graduates through direct survey. The objectives of this study were: (1) to determine whether CGSO fellowship graduates report sufficient preparedness for their careers; (2) to identify factors associated with readiness for practice following fellowship training; and (3) to pinpoint deficit areas in the current surgical oncology fellowship training model. We hope that this information will provide insight into the perception of readiness following CGSO fellowship and highlight areas for improvement to better meet the needs of practicing surgeons, the hospitals where they practice, and ultimately the patients in the community they serve.

## Methods

The Society of Surgical Oncology (SSO) leadership and National Cancer Institute (NCI) partnered to complete the survey study. Graduates were identified from a membership list provided by the SSO. The list included names and contact information (email addresses) for CGSO fellowship graduates from 2012 to 2022. We opted to survey graduates following the introduction of formal CGSO certification in 2012. From November 2022 to March 2023, the electronic survey was sent via Survey Monkey and directly distributed by the NCI and SSO to eligible graduates. The survey took approximately 8 min to complete. Participation was voluntary, and no financial incentives were offered. Invalid email addresses were excluded, and survey results were deidentified. The Office of Human Subjects Research Protections at the National Institutes of Health deemed the study exempt.

The survey consisted of 36 multiple choice, checkbox, dropdown, rank order, matrix, and fill-in-the-blank questions (Supplementary Appendix A). The survey was divided into the following sections: demographics, factors influencing CGSO fellowship selection, fellowship preparedness, and current practice patterns. The first part of the survey was designed to assess respondent demographics to determine whether underlying factors, such as type of surgical residency training program, research time, or previous locums and attending positions influenced preparedness. We also assessed reasons for choosing CGSO fellowship over other subspecialities. To assess the preparedness of CGSO fellowship graduates for practice, survey participants were asked about the extent to which the fellowship met their needs clinically, technically, and administratively. Additionally, graduates were asked whether there were any specific disease sites in which they felt unprepared. We also queried fellows on research preparedness; however, type of research (basic versus clinical versus translational) was not specifically assessed. The next portion of the survey focused on practice patterns. To understand the job market, graduates were asked to select one response on how long they were at their first position, how many jobs they interviewed at, how many offers were received, and how many jobs they held since fellowship. Our subsequent goal was to investigate whether the disease sites covered in fellowships are representative of those encountered by practicing surgeons. To determine the current practice patterns, we asked respondents their ideal breakdown, contract breakdown, and actual breakdown. They were asked to assign a percentage of 11 disease sites to total 100%.

## Results

The overall survey response rate was 38% (221/582) with male predominance (63%) (Table 1). The mean age of respondents was 40.4 years. Many respondents (24%) made the decision to pursue a CGSO fellowship during their PGY3 year, followed by PGY4 (21%), PGY2 (17%), before residency (16%), and PGY1 (14%). Sixty-five percent of graduates completed two or more years of research during residency, whereas 21% did not complete any research time. Nearly half (46%) of respondents completed their fellowship after 2019. Most fellows graduated from academic programs, either university hospital-based (75%) or community-based (19%). In terms of additional training or fellowships, 21% reported completing further training or fellowships beyond their CGSO fellowship, and a small proportion (9%) of graduates held attending or locum tenens positions prior to fellowship.

**Table 1:** Survey participant demographics

Characteristic	Respondents (*N* = 221)
Age (mean in years)	40.4
Unknown	3
Gender	
Man	138 (63%)
Woman	81 (37%)
Unknown	2
Year of CGSO Fellowship decision	
PGY1	29 (14%)
PGY2	36 (17%)
PGY3	51 (24%)
PGY4	44 (21%)
PGY5	11 (5.2%)
PGY6	3 (1.4%)
PGY7	5 (2.4%)
Before residency	33 (16%)
Unknown	9
Dedicated research years	
0	46 (21%)
1	30 (14%)
2	116 (53%)
3	19 (8.7%)
4+	7 (3.2%)
Unknown	3
Year of fellowship completion	
2012	16 (7.4%)
2013	14 (6.5%)
2014	13 (6.0%)
2015	18 (8.3%)
2016	22 (10%)
2017	18 (8.3%)
2018	15 (6.9%)
2019	20 (9.2%)
2020	30 (14%)
2021	24 (11%)
2022	25 (12%)
None of the above	2 (0.9%)
Unknown	4
Type of surgery residency	
Academic: community-based and university-affiliated	42 (19%)
Academic—university hospital-based	161 (74%)
Community—no university affiliation	6 (2.8%)
Military or federal facility	7 (3.2%)
Other (please specify)	2 (0.9%)
Unknown	3
Additional training	
No	172 (79%)
Yes	46 (21%)
Unknown	3
Previous experience	
Attending physician	15 (6.9%)
Locums position	6 (2.8%)
No	197 (90%)
Unknown	3

### Factors Influencing Fellowship Selection

**Fig. 1:**
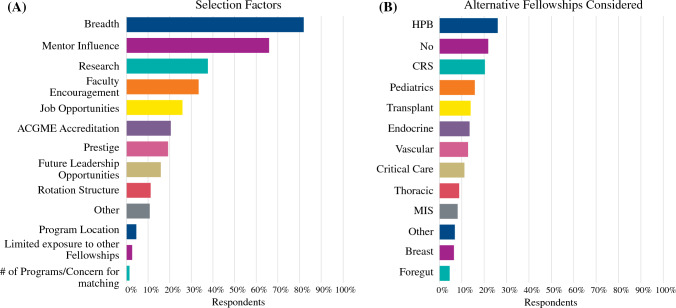
**A** CGSO fellowship selection factors. **B** Additional fellowships considered by graduates

According to survey responses, 46% of graduates decided to pursue surgical oncology before their third postgraduate year. Respondents were asked to select from several categories to determine which factors influenced their choice of fellowship. The breadth of cancer case exposure (82%), mentor influence (66%), and research opportunities (38%) were identified as the primary reasons for selecting CGSO over other subspecialties (Fig. 1A). Among the other fellowship options considered, graduates ranked HPB (26%), colon and rectal surgery (21%), and pediatric surgery (16%) as their top choices (Fig. 1B). Twenty-two percent of graduates did not consider any other fellowships.

### Preparedness for Practice

**Fig. 2:**
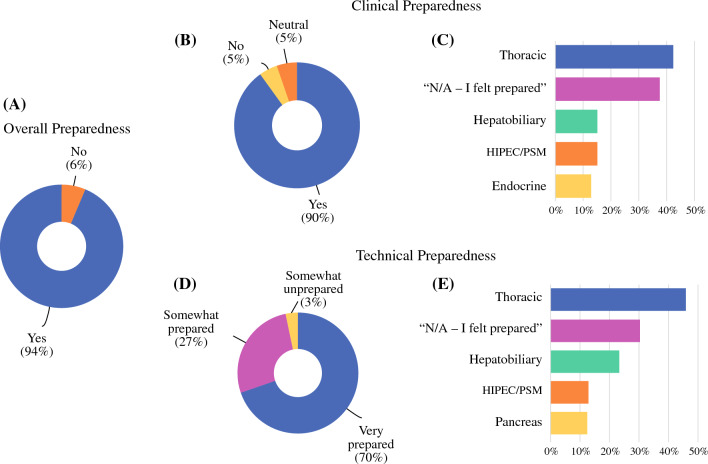
Did graduates feel prepared? **A** Overall, **B** Clinically, **C** For which disease sites did graduates report a lack of clinical preparedness? **D** Technically, **E** For which disease sites did graduates report a lack of technical preparedness?

**Fig. 3:**
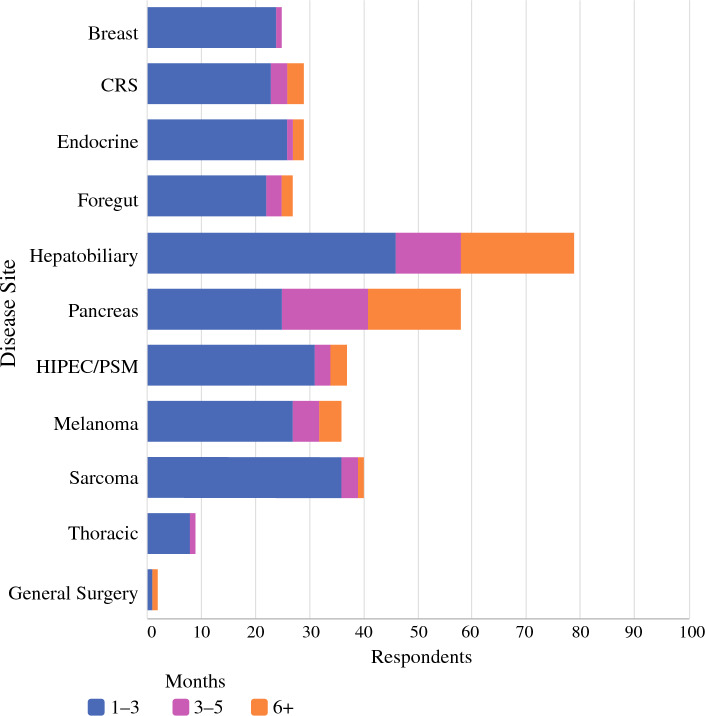
Where did fellows spend additional elective time?

Ninety-four percent of graduates report that they would be uncomfortable in their current practice without CGSO fellowship training (Fig. 2A). However, there were areas where fellows reported unpreparedness; 18% of respondents stated that they were unprepared or neutral in overall research (data not shown). Clinically, 90% of graduates reported overall preparedness (Fig. 2B), and 37% reported being prepared across all disease sites (Fig. 2C). The top four areas in which graduates reported feeling clinically unprepared were thoracic (43%), hepatobiliary (15%), HIPEC/peritoneal surface malignancy (PSM) (15%), and endocrine (13%) surgery. Respondents indicated they were technically very prepared (70%), somewhat prepared (27%), or somewhat unprepared (3%) (Fig. 2D). However, only 30% of graduates reported feeling technically prepared across all disease sites, whereas 46% reported being unprepared for thoracic surgery, 24% for hepatobiliary surgery, and 13% for HIPEC/PSM and pancreas surgery (Fig. 2E). Additional exposure to HPB on elective months during fellowship was common. Forty-nine percent of overall respondents reported spending additional elective time in liver surgery and 39% reported spending additional time in pancreas surgery (Fig. 3). Seven percent reported completing a formal HPB track. When assessing specific surgical approaches, respondents reported being unprepared in robotic (63%) and laparoscopic (33%) techniques.

According to graduates’ responses, they were administratively well-prepared (41%), somewhat prepared (46%), somewhat unprepared (9%), or not prepared (3%). Graduates suggested that increased autonomy (55%), program development (37%), increased case volumes (25%), and research infrastructure (24%) could improve training (data not shown).

### Post-fellowship Job Application Process

**Fig. 4:**
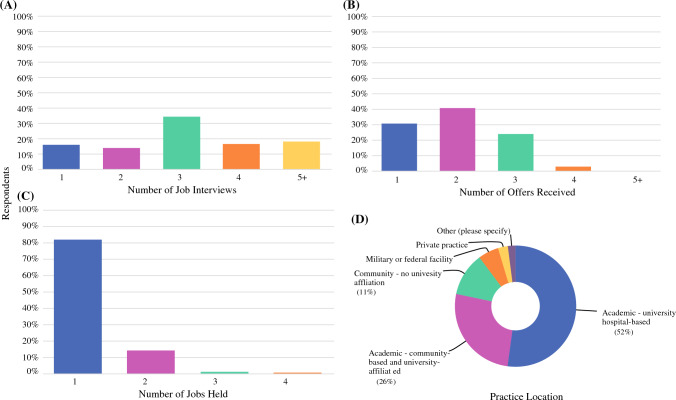
Job patterns of graduates. **A** Number of job interviews received, **B** Number of job offers received, **C** Number of jobs held since completing fellowship, **D** Current practice location

Median graduation year of survey respondents was 2018. Most (35%) graduates interviewed for three positions (median: 3.0); however, nearly 18% received 5+ interviews, and 16% received only one interview (Fig. 4A). Graduates reported receiving one, two, and three job offers at rates of 31%, 41%, and 24%, respectively (median: 2.0) (Fig. 4B). Those who received more interviews tended to have a greater number of job offers. Many graduates have been at their first position for 5 or more years (38%) (Fig. 4C), and those who received more job offers were more likely to remain in the same position since fellowship. CGSO graduates often practice at either academic, university hospital-based (52%) or academic, community-based affiliated (26%) hospitals (Fig. 4D). The remaining practice at other locations, such as community, miliary, or private-practice facilities.

### Post-fellowship Clinical Practice

**Fig. 5:**
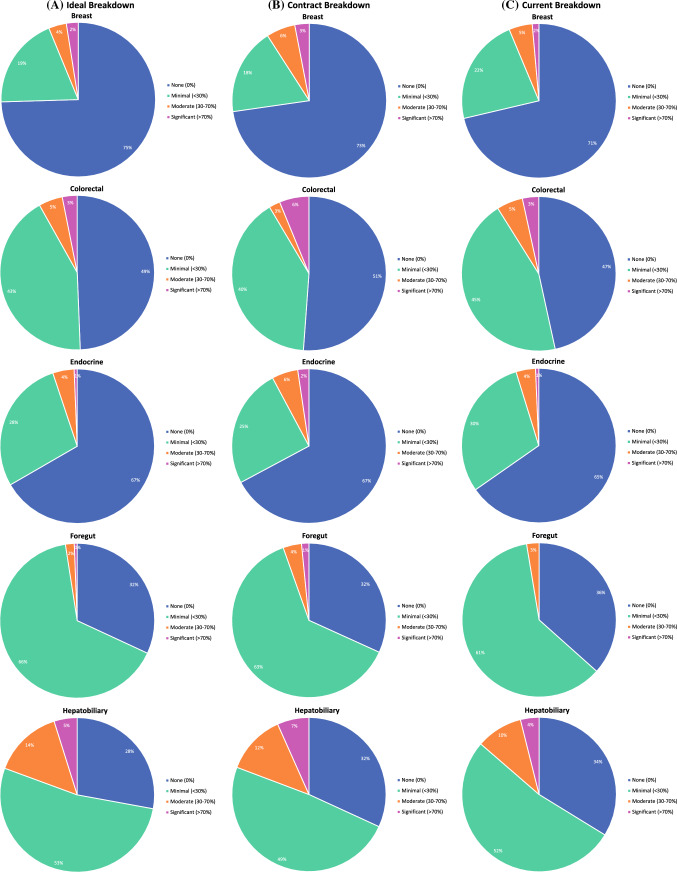

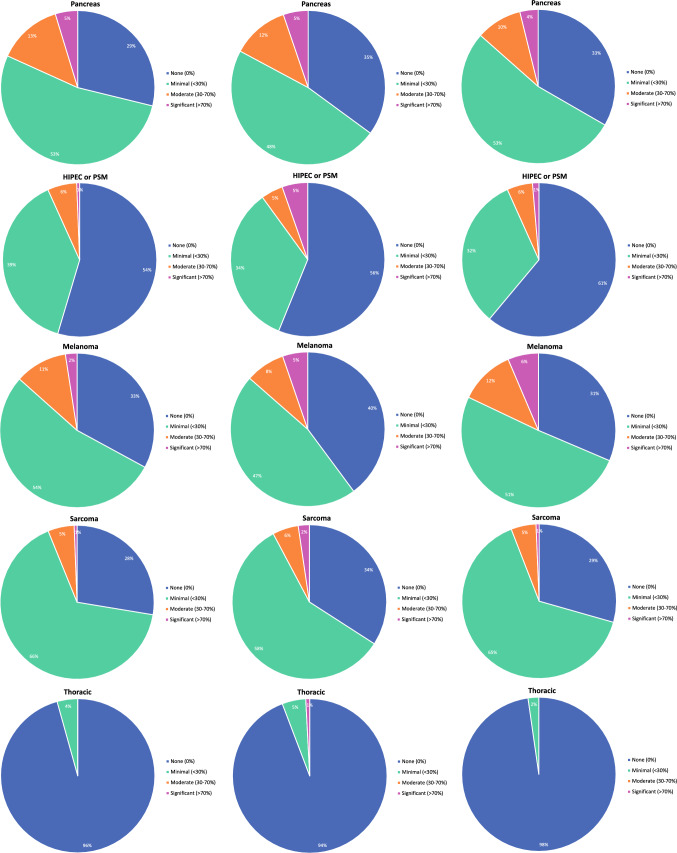

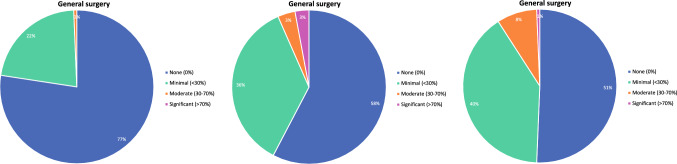
Percent composition of **A** Ideal, **B** Contract, **C** Current practice by disease site and by desired amount of time

Ideal, contract, and current clinical practice breakdowns for graduates by disease site are shown in Fig. 5. Hepatobiliary and pancreas were the most commonly reported sites in which graduates desired a significant portion of their ideal practice. In contrast, respondents reported that their ideal practice would not include breast, endocrine, thoracic, and general surgery (Fig. 5A). While 33% respondents desired a general surgery component in their practice, 42% reported their contract included general surgery, and 49% had general surgery in their current practice (Fig. 5). Nine percent of respondents indicated they spent at least a moderate portion of their time in general surgery (Fig. 5C). Approximately fifty percent of respondents’ contracts included a component of colorectal, foregut, hepatobiliary, pancreas, melanoma, and sarcoma (Fig. 5B). We also observed that while a greater number of graduates expressed an ideal practice that included hepatobiliary, pancreas, or HIPEC/PSM procedures, their actual practices incorporated fewer of these procedures than they indicated as desirable (Fig. 5).

**Fig. 6:**
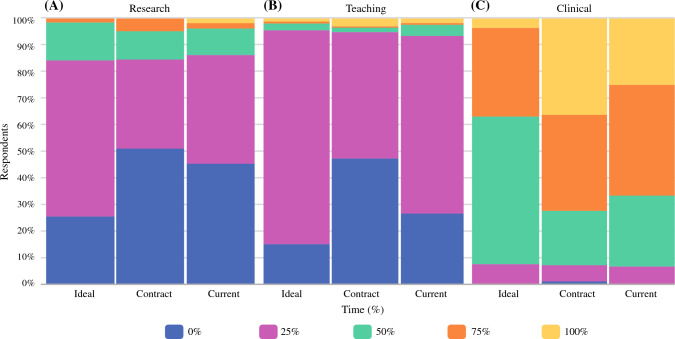
**A** Ideal, **B** Contract, **C** Current practice breakdown by research, teaching, and clinical duties

Lastly, 75% of respondents expressed a desire for research time as part of their practice (Fig. 6A). However, only about half of respondents reported receiving any research time, and a very small portion (3%) have a practice described as 100% research time. In terms of education, nearly 90% of respondents expressed an interest in at least some teaching time, typically constituting around a quarter of their contract breakdown (Fig. 6B). However, only 70% of respondents reported receiving teaching time, and 50% of graduates indicated that their contract had zero dedicated time. Regarding clinical practice, most graduates indicated a preference for dedicating 50–75% of their time to clinical work. In practice, however, a significant number of graduates end up spending 75–100% dedicated to clinical practice (Fig. 6C).

## Discussion

The creation of a CGSO fellowship was driven by the increasing complexity of cancer cases and the need for specialized training to provide high-quality, multidisciplinary care to cancer patients. However, the trainees’ perception of fellowship training to adequately prepare for a career in surgical oncology has not been assessed. Our study was designed to address this gap in the literature by assessing whether fellowship graduates reported sufficient preparedness for their careers, identifying factors associated with readiness for practice, and determining areas of improvement within the current CGSO fellowship model. Our findings suggest that CGSO fellowship effectively prepares graduates for careers in surgical oncology, as 94% of respondents reported that fellowship was essential to their current practice. However, we identified areas where graduates reported being unprepared and spent additional time. We also found discrepancies between trainee expectations and the realities of surgical oncology practice.

Overall, CGSO graduates reported being prepared for surgical oncology practice. Nevertheless, fellows highlighted various aspects in which they felt unprepared and needed to invest additional time. Nearly 10% of graduates completed formal HPB training, often requiring an extra year of fellowship. While a dedicated year of training provides additional experience in HPB, it necessitates an additional time commitment in an already lengthy training paradigm. This also raises an important point: there are limited combined CGSO-HPB training spots, and some graduates may end up practicing in settings where they would benefit from the additional training in this technically complex disease site. Additionally, prospective applicants may opt for the HPB fellowship instead of the broad-based CGSO fellowship if they feel it will better suit their needs. Furthermore, many graduates felt unprepared in HIPEC/PSM, despite some reporting spending additional elective time in this area. While it is not common for HIPEC/PSM to be the sole practice composition given its rarity, the question arises whether it would be advantageous to establish specialized paths within the CGSO fellowship to cater to their specific requirements.

While the majority of CGSO graduates practice in either academic hospital-based or academic community-based programs, there is a need for surgical oncologists to have broad-based training and address the need for surgical oncologists in more rural areas.^8^ Our study demonstrated that many graduates have a broad-based clinical practice, which is similar to previous reports.^9^ Additionally, we found that most residents who do a CGSO fellowship complete dedicated research time. Indeed, having a greater number of manuscript publications is associated with increased likelihood of matriculation into CGSO fellowship.^10,11^ However, our findings present a unique challenge for multiple reasons. Twenty-five percent of respondents in this survey did not want a practice with research, and the emphasis to complete dedicated research time may result in some CGSO fellowships passing over residents who do not have an “academic” track record. One potential solution would be to propose modifications in the current CGSO program requirements that allow certain existing programs (or new programs) to be more clinically focused or research focused based on a fellow’s ultimate career goals. This could allow certain programs to provide broad-based surgical oncology training, which may better prepare those who intend to practice in nontertiary centers. While some general surgical residency programs have implemented rural tracks, for example, additional tracking has not been done in CGSO fellowships.^12^ On the contrary, for those who desire to conduct research there may be a limited number of jobs that include dedicated research. Furthermore, nearly 20% of respondents reported not feeling adequately or neutrally prepared for a career in research. The current residency training paradigm incorporates dedicated research time during the second or third postgraduate years resulting in a substantial gap between research years and initial clinical practice. Furthermore, research options range from clinical, translational, and basic science. While generalized research instruction is a good starting point for fellowship programs, graduates may benefit from individualized research mentorship and education tailored to their ultimate career goals.

Over the past two decades, there have been significant changes in surgical training, including the implementation of duty hours and unintended decreased autonomy.^13^ Despite a rigorous 5-year surgical residency, 21% of program directors report that residents are not prepared for the operating room when entering fellowship.^14^ Our study, albeit a survey of the graduates not their educators, suggests that overall the CGSO fellowship prepares surgeons for practice. In the current study, the top areas graduates felt unprepared for included thoracic and hepatobiliary, which is consistent with a survey of graduating chief residents.^15^ Interestingly, many respondents in our survey felt that fellowship primarily provided multidisciplinary skills and knowledge, rather than technical skills and autonomy. While respondents suggested that increased autonomy and case volumes could enhance their preparation for practice, this may be a challenge at some select programs due to a limited number of cases distributed amongst a cohort of five to ten fellows per year. To overcome this barrier, some respondents suggested that fellowships should provide specialized and tailored training. However, this approach presents a challenge due to the constantly evolving job market, the availability of practice opportunities, and the need to satisfy ACGME Program Requirements and ABS requirements for board certification. Furthermore, our findings indicate that a significant number of graduates have a higher proportion of general surgery and broad-based surgical oncology in their practice than initially anticipated. This observation raises significant concerns about the potential impact of further subspecialization on the preparedness of graduates for the broad and diverse field of surgical oncology. Programs could consider dedicated elective months after fellows have job commitments, which would allow for increased exposure to disease sites specific to their proposed job.

The present study has limitations associated with cross-sectional survey studies. First, recall bias might affect the accuracy of graduates' self-reported preparedness. In addition, with a response rate of 38%, nonresponse bias could result in a nonrandom sample; however, this overall response rate is comparable to those in other surveys, and we observed similar baseline demographics among respondents. Moreover, there might be other factors influencing graduates’ reporting of preparedness that our study did not capture such as residency case numbers. Finally, because our primary objective was to assess the overall preparedness of graduates, we were unable to generalize the nuances of individual preparedness levels and determine readiness by training program. To further enhance our understanding of the effectiveness of CGSO fellowship, future research could be conducted by using a longitudinal approach. This could involve conducting a baseline survey of CGSO fellows before to the start of their fellowship, followed by several surveys at key points throughout the fellowship period, and then at intervals after the fellows have completed the program and entered the workforce. By tracking graduates over time, we would be able to gain information that could inform the development of more effective training programs.

## Conclusions

Complex General Surgical Oncology fellowships successfully develop comprehensive and competent surgical oncologists; however, some graduates may remain unprepared in specific clinical areas, resulting in additional elective time undertaken in these areas. The study demonstrates potential gaps between trainee expectations and the realities of surgical oncology practice. Our study suggests that CGSO fellowship programs may need to evolve to accommodate new surgical techniques and balance the provision of broad-based training while accommodating the need for specialized instruction in targeted disease sites for those who desire it. This approach will ensure that graduates emerge well-rounded while also having the opportunity to gain expertise in areas that are most relevant to their future careers.

### Supplementary Information

Below is the link to the electronic supplementary material.

Supplementary file1 (PDF 430 KB)
