# Supplementary material for: Perceptions of Readiness for Practice After Complex General Surgical Oncology Fellowship: A Survey Study
Source: Ann Surg Oncol. 2023 Nov 7;31(1):31–41. doi: 10.1245/s10434-023-14524-x (PMC10695882; doi:10.1245/s10434-023-14524-x)
Supplement: Supplementary file 1 — Supplementary file1 (PDF 430 KB) [file 10434_2023_14524_MOESM1_ESM.pdf]

1. Gender: How do you identify?

◯ Man

◯ Woman

◯ Non-binary

◯ Prefer not to answer

◯ Other (please specify)

[ ]

2. What is your current age?

[ ]

3. What year did you complete fellowship training?

[ ]

4. Which of the following best describes your general surgery residency program?

◯ Academic - university hospital-based

◯ Academic - community-based and university-affiliated

◯ Community - no university affiliation

◯ Military or federal facility

◯ Other (please specify)

[ ]

5. Did you complete any dedicated years of research during residency training?

- ◯ No
- ◯ 1
- ◯ 2
- ◯ 3
- ◯ 4+

6. Do you obtain an advanced degree prior to fellowship?

- ☐ No
- ☐ MPH
- ☐ MS
- ☐ PhD
- ☐ MBA
- ☐ Other (please specify)

7. Did you hold a locums or attending position prior to fellowship?

- ◯ No
- ◯ Locums
- ◯ Attending

8. Have you completed any additional training/fellowships?

- ◯ No
- ◯ Yes (comment below)

Additional training

These questions will help us determine why you chose CGSO over other specialties and determine if you felt adequately prepared for

your career.

9. During residency, which year did you decide on CGSO?

10. During residency, did you consider other subspecialty fellowships?

- [ ] No
- [ ] Breast
- [ ] Colorectal
- [ ] Endocrine
- [ ] Foregut
- [ ] HPB
- [ ] MIS
- [ ] Pediatric
- [ ] Surgical Critical Care
- [ ] Thoracic
- [ ] Transplant
- [ ] Vascular
- [ ] Other (please specify)

11. What impacted your selection of a CGSO fellowship **over** any other fellowship (select all that apply)?

- [ ] Employment opportunities
- [ ] Future leadership opportunities
- [ ] Breadth of cancer case exposure
- [ ] Research opportunities
- [ ] Program location
- [ ] Prestige or program reputations
- [ ] Number of programs and applicants/concern for ability to match
- [ ] Guaranteed formal rotations in medical oncology, radiation oncology, and pathology
- [ ] Limited exposure to other fellowships
- [ ] Residency faculty encouraged CGSO fellowship
- [ ] ACGME accreditation of fellowship and/or ABS eligibility for certification
- [ ] Mentor influence
- [ ] Other (please specify)

12. Did fellowship meet your research training needs for your current practice?

- ○ Yes
- ○ No
- ○ Neutral

13. Do you believe your fellowship met your clinical (overall patient clinical care) needs for your current practice?

- ◯ Yes
- ◯ No
- ◯ Neutral

14. Please select any specific disease site areas where you felt unprepared **clinically**?

- ☐ Breast
- ☐ Colorectal
- ☐ Endocrine
- ☐ Foregut
- ☐ Hepatobiliary
- ☐ Pancreas
- ☐ HIPEC or Peritoneal Surface Malignancy (PSM)
- ☐ Melanoma
- ☐ Sarcoma
- ☐ Thoracic
- ☐ General surgery
- ☐ None - I felt prepared clinically in all areas

Other (please specify)

15. Did you feel prepared **technically** after fellowship training?

- ◯ Very prepared
- ◯ Somewhat prepared
- ◯ Somewhat unprepared
- ◯ Not prepared

16. Please select any specific disease site areas where you felt unprepared **technically**?

- ☐ Breast
- ☐ Colorectal
- ☐ Endocrine
- ☐ Foregut
- ☐ Hepatobiliary
- ☐ Pancreas
- ☐ HIPEC or Peritoneal Surface Malignancy (PSM)
- ☐ Melanoma
- ☐ Sarcoma
- ☐ Thoracic
- ☐ General surgery
- ☐ N/A - I felt prepared technically in all areas
- ☐ Other (please specify)

17. In which surgical areas did you feel unprepared?

- ☐ Open technique operations
- ☐ Laparoscopic technique operations
- ☐ Robotic operations
- ☐ Outpatient clinic procedures
- ☐ Other (please specify)

18. Did you feel prepared **administratively** after fellowship training? (Documentation, understanding faculty roles, etc.)

- ○ Very prepared
- ○ Somewhat prepared
- ○ Somewhat unprepared
- ○ Not prepared

19. What aspects of your training could improve to better to prepare you?

- ☐ Higher case volume
- ☐ More diverse case exposure
- ☐ Increased autonomy in OR
- ☐ Improved mentorship
- ☐ Dedicated education in clinical care/decision making
- ☐ Increased training in program development
- ☐ Improved research infrastructure/mentorship

20. Given the composition of your current practice, would you feel comfortable doing your current cases **without** any fellowship training?

- ◯ Yes
- ◯ No

Would you please explain your answer?

21. Do you feel the current length of CGSO fellowship is appropriate?

- ◯ Yes
- ◯ No – Should be shorter
- ◯ No – Should be longer

22. If you had to the opportunity to choose again, would you have chosen the same pathway?

- ◯ Yes
- ◯ No

These questions will help us obtain insight into your current

practice to identify potential deficits in the current training model.

23. How long have you been/were you at your first position? (in years)

- ◯ <1
- ◯ 2
- ◯ 3
- ◯ 4
- ◯ 5+

24. How many jobs did you interview for your first position?

- ◯ 1
- ◯ 2
- ◯ 3
- ◯ 4
- ◯ 5+

25. When interviewing for your first job, how many job offers did you receive?

- ◯ 1
- ◯ 2
- ◯ 3
- ◯ 4
- ◯ 5+

26. How many jobs have you held since completing surgical oncology fellowship?

- ◯ 1
- ◯ 2
- ◯ 3
- ◯ 4
- ◯ 5+

27. Which of the following most closely describes your first position?

- ◯ Academic - university hospital-based
- ◯ Academic - community-based and university-affiliated
- ◯ Community - no university affiliation
- ◯ Military or federal facility
- ◯ Private practice
- ◯ Rural practice
- ◯ Other (please specify)

28. Did you spend additional time (ex. an elective) during fellowship in any of the following areas?

	None	1-3 months	3-5 months	6+ months
Breast				
Colorectal				
Endocrine				
Foregut				
Hepatobiliary				
Pancreas				
HIPEC or PSM				
Melanoma				
Sarcoma				
Thoracic				
General surgery				

29. What is/was the **ideal** breakdown of your practice in each of the following areas?

	0%	10%	20%	30%	40%	50%	60%	70%	80%	90%	100%
Breast											
Colorectal											
Endocrine											
Foregut											
Hepatobiliary											
Pancreas											
HIPEC or PSM											
Melanoma											
Sarcoma											
Thoracic											
General surgery											

30. What was your **contract** breakdown for practice in each of the following areas?

	0%	10%	20%	30%	40%	50%	60%	70%	80%	90%	100%
Breast											
Colorectal											
Endocrine											
Foregut											
Hepatobiliary											
Pancreas											
HIPEC or PSM											
Melanoma											
Sarcoma											
Thoracic											
General surgery											

31. What is the **current** breakdown of your practice in each of the following areas?

	0%	10%	20%	30%	40%	50%	60%	70%	80%	90%	100%
Breast											
Colorectal											
Endocrine											
Foregut											
Hepatobiliary											
Pancreas											
HIPEC or PSM											
Melanoma											
Sarcoma											
Thoracic											
General surgery											

32. What is/was your **ideal** breakdown in your career? (On average, what percentage of each week would you spend in each of the following activities?)

	0%	25%	50%	75%	100%
Research	○	○	○	○	○
Teaching	○	○	○	○	○
Clinical	○	○	○	○	○
Other extra-curricular (ex. quality and safety, administrative, etc.)	○	○	○	○	○

33. When hired for your current job, what was the **contract** breakdown of the following activities? (On average, what percentage of each week was planned in each of the following activities?)

	0%	25%	50%	75%	100%
Research	○	○	○	○	○
Teaching	○	○	○	○	○
Clinical	○	○	○	○	○
Other extra-curricular (ex. quality and safety, administrative, etc.)	○	○	○	○	○

34. What is the **current** breakdown of activities at your current job? (On average, what percentage of each week do you spend in each of the following activities?)

	0%	25%	50%	75%	100%
Research	○	○	○	○	○
Teaching	○	○	○	○	○
Clinical	○	○	○	○	○
Other extra-curricular (ex. quality and safety, administrative, etc.)	○	○	○	○	○

35. What is your **ideal** practice 3 years from now?

- [ ] The same practice I currently have
- [ ] Less clinical time
- [ ] More clinical time
- [ ] Less research time
- [ ] More research time
- [ ] Less education time
- [ ] More education time
- [ ] Less extracurricular activities
- [ ] More extracurricular activities
- [ ] More administrative work
- [ ] Less administrative work
- [ ] Other (please specify)

36. If you have any additional comments to your satisfaction with fellowship training, please include below
